# Energy Drinks, Alcohol, Sports and Traumatic Brain Injuries among Adolescents

**DOI:** 10.1371/journal.pone.0135860

**Published:** 2015-09-16

**Authors:** Gabriela Ilie, Angela Boak, Robert E. Mann, Edward M. Adlaf, Hayley Hamilton, Mark Asbridge, Jürgen Rehm, Michael D. Cusimano

**Affiliations:** 1 Injury Prevention Research Office, St. Michael’s Hospital, Toronto, Canada; 2 Department of Psychology, University of Toronto, Toronto, Canada; 3 Social and Epidemiological Research, Centre for Addiction and Mental Health, Toronto, Canada; 4 Dalla Lana School of Public Health, University of Toronto, Toronto, Canada; 5 Dalhousie University, Halifax, Canada; 6 Division of Neurosurgery, University of Toronto, Toronto, Canada; Hospital Nacional de Parapléjicos - SESCAM, SPAIN

## Abstract

**Importance:**

The high prevalence of traumatic brain injuries (TBI) among adolescents has brought much focus to this area in recent years. Sports injuries have been identified as a main mechanism. Although energy drinks, including those mixed with alcohol, are often used by young athletes and other adolescents they have not been examined in relation to TBI.

**Objective:**

We report on the prevalence of adolescent TBI and its associations with energy drinks, alcohol and energy drink mixed in with alcohol consumption.

**Design, Settings and Participants:**

Data were derived from the Centre for Addiction and Mental Health’s 2013 Ontario Student Drug Use and Health Survey (OSDUHS). This population-based cross-sectional school survey included 10,272 7^th^ to 12^th^ graders (ages 11–20) who completed anonymous self-administered questionnaires in classrooms.

**Main Outcome Measures:**

Mild to severe TBI were defined as those resulting in a loss of consciousness for at least five minutes, or being hospitalized for at least one night. Mechanism of TBI, prevalence estimates of TBI, and odds of energy drink consumption, alcohol use, and consumption of energy drinks mixed with alcohol are assessed.

**Results:**

Among all students, 22.4% (95% CI: 20.7, 24.1) reported a history of TBI. Sports injuries remain the main mechanism of a *recent* (past year) TBI (45.5%, 95% CI: 41.0, 50.1). Multinomial logistic regression showed that relative to adolescents who never sustained a TBI, the odds of sustaining a *recent* TBI were greater for those consuming alcohol, energy drinks, and energy drinks mixed in with alcohol than abstainers. Odds ratios were higher for these behaviors among students who sustained a *recent* TBI than those who sustained a *former* TBI (lifetime but not past 12 months). Relative to recent TBI due to other causes of injury, adolescents who sustained a recent TBI while playing sports had higher odds of recent energy drinks consumption than abstainers.

**Conclusions and Relevance:**

TBI remains a disabling and common condition among adolescents and the consumption of alcohol, energy drinks, and alcohol mixed with energy drinks further increase the odds of TBI among adolescents. These associations warrant further investigation.

## Introduction

In recent years, traumatic brain injuries (TBI) among children and adolescents have been identified as a major health concern in the United States and Canada, with increases of 57% in sports-related TBI in the US between 2001 and 2009 [1–5]. Recently, we reported that 5.6% of students in grades 7–12 in Ontario, Canada reported a TBI in the previous 12 months, most of which were identified as having occurred during team sports play [5]. Sports offer youth many health benefits, and a recent report notes that only 4% of 12- to 17-year-olds in Canada meet the recommended guidelines of 60 minutes of moderate to vigorous activity daily, while North-American youth 12 to 17-years-old spend an average of 9.3 hours daily being sedentary [6]. Nevertheless, rates of sports-related TBI among adolescents have been described as an epidemic [7]. Many of these injuries are unreported and are not considered serious by youth, their parents, coaches and often medical practitioners [8]. Positive attitudes towards violence in team sports (e.g., hockey, American football) embedded in North American culture may also play a role in this problem [9]. The Centre for Disease Control and Prevention and the Institute of Medicine have recommended several actions to address this problem, including research on the risks and consequences of TBI, and knowledge translation to the public and the vulnerable populations (e.g., young and old athletes) [1–5].

One in five adolescents in a 2011 Canadian sample reported sustaining a traumatic brain injury in their lifetime [5]. Adolescent TBI raises concerns given the acute and chronic cognitive, emotional and psychosocial consequences such injured adolescent may endure during a period in their development (when social skills are emerging, in a context of increasing environmental demands) [5,7,10–13]. The negative social consequences of TBI during adolescence are further illustrated by the elevated prevalence of TBI among criminal and delinquent populations and evidence that links TBI to violence and criminal acts [14–16]. We recently observed strong relationships among a history of TBI and substance misuse, mental health problems, suicidality, violent conduct behaviours, bullying and poor academic performance in a 2011 population sample of Ontario adolescents [5,10–11].

Energy drink consumption among adolescents in Canada and the US has been increasing recently and evidence suggests that energy drink consumption is linked with playing sports (a main mechanism of TBI among adolescents) and being male (the sex reporting most TBI history among adolescents) [17–20]. In 2010 approximately 16% more energy drinks were sold than the previous year with sales close to $9 billion dollars in the US, and in Ontario, Canada in 2013 almost 40% of adolescents consumed an energy drink in the past year [21–22]. Energy drink consumption among adolescents is associated with injury and other adverse correlates, and existing research suggests that adolescents who are attracted to energy drink consumption are generally engaged in more risk behaviours and experience greater adverse outcomes than non-energy drink consumers [17–24]. The role of the pharmacological effects of the energy drinks over systematic consumer characteristics in perpetuating adverse outcomes, however, is yet to be clarified [20–21].

To our knowledge, no study to date has examined the possible association between energy drink consumption and TBI among adolescents. The combination of TBI with energy drinks consumption, or TBI with alcohol and energy drinks consumption may complicate and lengthen recovery efforts, since it is not uncommon for TBI individual’s life to continue to improve many years after the brain injury [12]. Given that the brain is still developing during adolescence, trauma to the head, in addition to drinking alcohol with energy drinks, or consuming high levels of energy drinks post injury may decrease or interfere with the chance of improving post injury, or may predispose the individual to higher risk of having another brain injury [12]. Thus an examination of how a TBI and energy drinks consumption, and energy drinks with alcohol relate is important to medical professionals, parents, sports coaches, and school guidance councilors to aid the understanding and prevention efforts, but also for diagnosis and the recovery planning of adolescents with TBI, particularly since even mild forms of TBI may have disabling consequences [4–5,10–13,15–16,25–26]. In this paper we describe the influence of the consumption of alcohol, energy drinks, and energy drinks mixed with alcohol, on the odds of sustaining a recent or former TBI, and recent sports related TBI in a population sample of adolescents in Ontario, Canada.

## Methods

The study was approved by the Research Ethics Committees of the Centre for Addiction and Mental Health, St. Michael’s Hospital, participating Ontario Public and Catholic school boards, and York University, which administered the surveys. The study was conducted according to the principles expressed in the *Declaration of Helsinki*. All participants provided their signed assent in addition to parentally signed consent for those aged under 18.

Data were based on 10,272 7^th^-12^th^ graders (ages 11–20) and were derived from the Centre for Addition and Mental Health’s (CAMH) 2013 Ontario Student Drug Use and Health Survey (OSDUHS), a repeated cross-sectional probability survey of Ontario students enrolled in provincially funded schools. The sample excluded private, military, institutional and low enrolment schools, and special education, English as a second language and low enrolment classes. These exclusions represent a small proportion of the Ontario student population (about 8%). Therefore, although our target population represents students, it captures the vast majority (92%) of Ontario children and adolescents aged 12–18 years.

Students completed a self-administered, anonymous pen-and-paper questionnaires in their classrooms between November 2012 and June 2013. The school, class and student participation rates were 61%, 87% and 63%, respectively. A comparison between high responding and low responding classes showed no evidence of nonresponse bias for a set of health-relate behaviours [27]. The 2013 OSDUHS employed a stratified (region by school type [elementary, secondary]), two-stage (school, class) cluster sample design. Within each strata, schools were selected with probability-proportional-to-size, and within selected schools, classes were selected with equal probability. Students completed one of two alternately distributed (i.e., A, B, A) questionnaires (Form A or Form B). The TBI, alcohol, energy drinks, sex, gender and current marks in school items were found in both forms A and B which were answered by 10,272 students drawn from 198 schools and 671 classes dispersed province wide. Form B exclusively contained the energy drinks mixed with alcohol question and was answered by a half sample of 4,794 students. Detailed description of the survey methods, including the sampling design and how the survey is administered within schools and classes, is web-available [27].

### Measures

*Traumatic brain injury* (TBI) was defined to students as a blow or a hit to the head that rendered the student unconscious for at least five minutes or resulted in their hospitalization for at least one night. This criteria is also employed in several classification systems including DSM-IV and has previously been used in adolescent and adult studies [28–32]. Students were asked if they ever had such injury in the 12 months prior (*recent TBI*) or in their lifetime, but not past 12 months (*former TBI*). Students reporting a recent TBI in the 12 months prior were asked about the source of the injury. To assess potential nonresponse bias, we compared high-participating classes (those with 70% or more of students in the class participating) to low-participating classes (less than 70% participating) in their TBI responses, and found no evidence of nonresponse bias (for former TBI: 21.5% vs. 22.4%, *t*_669_ = -0.889, *P* = 0.374; and recent TBI: 6.3% vs. 6.1%, *t*_669_ = 0.344, *P* = 0.731).

*Mechanism of TBI* was assessed in the survey for past 12 months TBI reports only, and was coded 0 (labeled other causes of injury) for motor vehicle accident, other vehicle accidents, bicycle accident, fell down by accident, fight, bullied (pushed by someone, and other causes not listed, and 1 for sports injuries.

The *alcohol* question read: “In the last 12 months, how often did you drink alcohol—liquor (rum, whiskey, etc.), wine beer, coolers?” Data were coded 1, labelled “*never*” (for “never drank alcohol in my lifetime”); were coded 2, labelled “*lifetime*, *excluding past 12 months*” (for “drank alcohol before, but not in the last 12 months”); were coded 3, labelled “*infrequent drinking*” (for “had a sip of alcohol to see what it’s like” or “drank only at special events such as holidays or weddings”); and were coded 4, labelled “*occasionally/frequent drinking*” (for “once a month or less often”, “2 or 3 times a month”, “once a week”, “2 or 3 times a week”, “4 or 5 times a week”, or “almost every day”).

*Energy drink consumption* was assessed with a question that asked students how often they drank “a can of a high-energy caffeine drink, such as Redbull, Rockstar, Full Throttle, Monster, etc.” in the past 12 months? Response categories referred to consumption in the last 7 days contrasting among 1, labeled “never”, for no consumption in the last 7 days or in the last 12 months; 2, labeled “*past 12 months”*, for no consumption in the last 7 days, but some consumption in the last 12 months; 3, labeled *“once in the past 7 days*”; 4, labeled *“2–4 times in the past 7 days”* for 2 to 4 times; 5, labeled *“5+ in the past seven days”*, for consumption that exceeded 5 times in the past 7 days.

Students’ consumption of *alcohol mixed with an energy drink* was assessed by the following question: “In the last 12 months, how often did you drink an energy drink mixed with alcohol, such as Red Bull mixed with alcohol, Rock Star + vodka, or other brands?” The options were 1, labeled *“never”*, for never drank an energy drink with alcohol in lifetime, 2, labeled *“lifetime”* for drank an energy drink with alcohol, but not in the last 12 months; 3, labeled *“1–2 times”*, for 1 or 2 times; 4, labeled *“3–5 times”* for 3 to 5 times; 5, labeled *“6+ times”* for more than 6 times in the past 12 months. From a policy prospective, it is important to note that this definition refers to premixed and hand-mixed beverages, as opposed to alcohol and energy drink consumed within the same drinking session.

We included *academic performance* as a covariate based on earlier work showing an association between TBI status and academic performance 5]. Academic performance was defined as marks (on average) currently obtained in school. The variable was coded (1) 90–100%; (2) 80–89%; (3) 70–79%; (4) 60–69%; and (5) below 60%.

### Analysis

Because data derived from complex surveys using stratification and clustering underestimate variances (and thus overstate significance levels) analyses must employ design based estimation methods. All analyses employed a complex sample design with 20 strata (region by school level), and 198 primary sampling units (schools) estimated by Taylor series linearization (TSL) executed in the Complex Sample module in SPSS version 22.0 (SPSS Inc., 2013). Logistic regressions predicting membership of those sustaining a recent and former TBI status (vs. never sustaining a TBI), and were performed fitting six factors—sex, grade (7 through 12), past-year alcohol use, past year alcohol-energy drink consumption, past year energy drink consumption, and academic performance, against P < 0.05 (two-tailed). We performed three multinomial logistic regression one for each of the three factors: past-year alcohol use, energy drinks and energy drinks mixed with alcohol, while holding fixed sex and academic performance, against a two-tailed *P* < 0.05. We fit single regression models for each factor to reduce multicollinearity which posses difficulties for the estimation of multinomial logistic models [33]. Listwise deletion reduced the estimation sample to 10,102 from 10,272 (for the entire sample), and to 4670 from 4794.

## Results

Table 1 describes demographic characteristics and the mechanisms of injury. The estimated lifetime prevalence of TBI among Ontario adolescents was 22.4% (95% CI: 20.7, 24.1); 6.0% (95% CI: 5.1, 7.1) reported at least one *recent* TBI (in the past 12 months) and 16.3% (95% CI: 15.1, 17.3) reported a *former* TBI (in their lifetime but not in the past 12 months). Sports injuries accounted for almost half of the recent TBI cases (45.5% [95% CI: 41.0, 50.1]) and were more common among males than females (51.8% vs. 34.7%, respectively).

**Table 1:** Weighted and unweighted estimates of TBI by demographic characteristics and mechanisms of injury for Ontario 7^th^–12^th^ graders, 2013 OSDUHS (N = 10,102).

	Sample characteristics	Never	Lifetime^a^	Past 12 months	
	% (95% CI)	% (95% CI)	% (95% CI)	% (95% CI)	
	(n = 10102)	(n = 7904)	(n = 1575)	(n = 623)	
**Sex**					
***Females (n)***	(5546)	(4558)	(721)	(267)	
*Weighted*	*48*.*4 (46*.*2*, *50*.*6)*	*81*.*9 (80*.*2*, *83*.*5)*	*13*.*1 (11*.*9*, *14*.*5)*	*5*.*0 (4*.*0*, *6*.*1)*	
Unweighted	54.9 (53.5, 56.3)	82.2 (81.1, 83.2)	13.0 (12.2, 13.9)	4.8 (4.2, 5.5)	
*Mechanism of Injury*				*Sports injury*	*34*.*7 (28*.*4*, *41*.*5)* ^d^
					34.0 (29.7, 38.6)^e^
				*Fell down by accident*	*22*.*3 (17*.*0*, *28*.*7)* ^d^
					21.2 (17.9, 24.9)^e^
				*Fight*	*2*.*3 (0*.*9*, *5*.*7)* ^d^
					1.9 (1.1, 3.3)^e^
				*Bullied (pushed)*	*2*.*5 (1*.*2*, *5*.*2)* ^d^
					3.0 (1.9, 4.5)^e^
				*MVA* ^b^	*2*.*6 (1*.*2*, *5*.*9)* ^d^
					3.8 (2.5, 5.7)^e^
				*Other VA* ^c^	*1*.*5 (0*.*5*, *4*.*3)* ^d^
					1.9 (1.1, 3.4)^e^
				*Bicycle accident*	*3*.*5 (1*.*6*, *7*.*2)* ^d^
					3.3 (2.1, 5.1)^e^
				*Other causes*	*30*.*6 (24*.*3*, *37*.*8)* ^d^
					30.9 (27.0, 35.1)^e^
***Males (n)***	(4556)	(3346)	(854)	(356)	
*Weighted*	*51*.*6 (49*.*6*, *53*.*8)*	*74*.*1 (71*.*4*, *76*.*6)*	*19*.*0 (17*.*0*, *21*.*2)*	*6*.*9 (5*.*7*, *8*.*3)*	
Unweighted	45.1 (43.7, 46.5)	73.4 (72.0, 74.8)	18.7 (17.5, 20.0)	7.8 (7.0, 8.7)	
*Mechanism of Injury*				*Sports injury*	*51*.*8 (45*.*7*, *57*.*9)* ^d^
					51.0 (46.6, 55.3)^e^
				*Fell down by accident*	*12*.*0 (8*.*5*, *16*.*6)* ^d^
					13.1 (10.6, 16.1)^e^
				*Fight*	*4*.*4 (2*.*5*, *7*.*7)* ^d^
					4.3 (3.0, 6.2)^e^
				*Bullied (pushed)*	*2*.*9 (1*.*1*, *7*.*6)* ^d^
					1.5 (0.8, 2.8)^e^
				*MVA* ^d^	*3*.*9 (1*.*1*, *13*.*2)* ^d^
					2.2 (1.3, 3.5)^e^
				*Other VA* ^e^	*2*.*7 (1*.*5*, *5*.*1)* ^d^
					2.4 (1.6, 3.8)^e^
				*Bicycle accident*	*1*.*4 (3*.*4*, *9*.*2)* ^d^
					6.1 (4.6, 8.1)^e^
				*Other causes*	*16*.*6 (12*.*6*, *21*.*4)* ^d^
					19.4 (16.6, 22.4)^e^
**Grade 7 (n)**	(2034)	(1587)	(320)	(127)	
*Weighted*	*11*.*7 (9*.*9*, *13*.*9)*	*78*.*6 (75*.*3*, *81*.*6)*	*17*.*4 (15*.*2*, *19*.*9)*	*4*.*0 (2*.*6*, *6*.*0)*	
Unweighted	20.1 (18.9, 21.5)	78.0 (75.9, 80.0)	15.7 (14.2, 17.2)	6.2 (5.2, 7.5)	
**Grade 8 (n)**	(1978)	(1566)	(281)	(131)	
*Weighted*	*12*.*6 (10*.*5*, *15*.*1)*	*79*.*1 (74*.*9*, *82*.*7)*	*14*.*9 (11*.*4*, *19*.*4)*	*6*.*0 (4*.*5*, *8*.*0)*	
Unweighted	19.6 (18.2, 21.0)	79.2 (77.1, 81.1)	14.2 (12.5, 16.1)	6.6 (5.5, 7.9)	
**Grade 9 (n)**	(1523)	(1176)	(247)	(100)	
*Weighted*	*16*.*6 (15*.*2*, *18*.*1)*	*77*.*1 (74*.*3*, *79*.*7)*	*15*.*0 (12*.*5*, *17*.*9)*	*7*.*9 (6*.*0*, *10*.*3)*	
Unweighted	15.1 (13.4, 16.9)	77,2 (75.1, 79.2)	16.2 (14.5, 18.1)	6.6 (5.4, 8.0)	
**Grade 10 (n)**	(1524)	(1189)	(252)	(83)	
*Weighted*	*16*.*9 (15*.*5*, *18*.*4)*	*79*.*3 (76*.*2*, *82*.*1)*	*16*.*2 (13*.*4*, *19*.*5)*	*4*.*4 (3*.*2*, *6*.*2)*	
Unweighted	15.1 (13.8, 16.4)	78.0 (75.8, 80.1)	16.5 (14.6, 18.6)	5.4 (4.4, 6.7)	
**Grade 11 (n)**	(1554)	(1214)	(249)	(91)	
*Weighted*	*17*.*9 (16*.*6*, *19*.*3)*	*77*.*3 (73*.*2*, *81*.*0)*	*16*.*1 (13*.*4*, *19*.*2)*	*6*.*6 (4*.*6*, *9*.*3)*	
Unweighted	15.4 (13.8, 17.1)	78.1 (75.6, 80.4)	16.0 (14.4, 17.8)	5.9 (4.6, 7.4)	
**Grade 12 (n)**	(1489)	(1172)	(226)	(91)	
*Weighted*	*24*.*3 (22*.*3*, *26*.*4)*	*76*.*8 (73*.*0*, *80*.*3)*	*17*.*0 (14*.*2*, *20*.*2)*	*6*.*2 (4*.*0*, *9*.*4)*	
Unweighted	14.7 (13.5, 16.1)	78.7 (76.4, 80.8)	15.2 (13.4, 17.2)	6.1 (5.0, 7.5)	

Note:

^a^ Lifetime, excluding past 12 months;

^b^ Motor vehicle accident;

^c^ Other vehicle accident

^d^ Weighted estimates;

^e^ Unweighted estimates

Our preliminary logistic regression (not tabled) identified sex, alcohol use, energy drinks, energy drinks mixed with alcohol and academic performance as significant predictors of TBI. Because level grade was not significantly associated with TBI status we excluded it from further analyses [33]. Relative to students who never sustained a TBI, the odds of sustaining a former TBI were significantly greater among students who obtained grades, on average, lower than 60% (OR = 3.43, 95% CI: 1.33, 8.83), between 60 to 69% (OR = 2.59, 95% CI: 1.83, 3.67), and between 70 to 79% (OR = 1.94, 95% CI: 1.48,2.56) than those with grades 90% or above. Relative to students who never sustained a TBI, the odds of sustaining a recent TBI were significantly greater among students with grades below 60% (OR = 5.88, 95% CI: 1.66, 20.79), and between 60–69% (OR = 2.80, 95% CI: 1.24, 6.34) than those with grades at or above 90%. Therefore subsequent regressions held constant academic performance and sex.

The three multinomial logistic regressions reliably distinguished among the three TBI groups (former, recent, never) for all three predictors compared to abstainers (Table 2); alcohol use Wald’s *F (10*,*169)* = 14.74, P < 0.001, energy drinks consumption Wald’s *F (12*,*167)* = 29.25, P < 0.001, and alcohol-energy drink consumption Wald’s *F (12*,*154)* = 10.33, P < 0.001. There are two general features evident in this analysis. First, the odds of sustaining a former TBI or recent TBI increase with the consumption of both alcohol and energy drinks. This pattern is not evident for alcohol-energy drink consumption on former TBI. Second, the odds of sustaining a former TBI or recent TBI are greater for sustaining a recent TBI rather than a former TBI. Indeed, the increasing ORs are appreciably greater for those sustaining a recent TBI, than a former TBI.

**Table 2:** Multinomial Logistic Regression analyses predicting *former* (lifetime but not in the past 12 months) and *recent* (past 12 months) TBI by fitting alcohol use, energy drinks, energy drinks mixed with alcohol, respectively, while controlling for sex and academic performance. The Table displays adjusted Odds Ratios (aOR) and design adjusted 95% CI for TBI, 2013 OSDUHS.

	Former TBI vs. Never	Recent TBI vs. Never
	aOR (95% CI)	aOR (95% CI)
**Alcohol Use** ^b^ **(n = 9899)** ^1^	F (6,173) = 16.25***	
Never used	1.00 (Reference)	1.00 (Reference)
Lifetime^a^	1.53 (.87, 2.70)	1.53 (.58, 4.01)
Infrequently^b^	1.29 (1.05, 1.60)*	1.98 (1.33, 2.95)**
Occasionally/Frequently^c^	1.54 (1.21, 1.96)**	3.65 (2.59, 5.14)***
*Sex* ^d^	F (2,177) = 9.76***	
*Current marks in school*, *on average* ^d^	F (2,177) = 19.25***	
**Energy drinks** ^b^ **(n = 9969)** ^2^	F (8,171) = 15.38***	
Never^b^	1.00 (Reference)	1.00 (Reference)
Past 12 months	1.55 (1.26, 1.91)***	2.04 (1.45, 2.87)***
Once in past 7 days	2.34 (1.63, 3.37)***	5.57 (3.23, 9.62)***
2–4 times in past 7 days	2.24 (1.39, 3.61)**	4.73 (2.57,8.68)***
5 + in past 7 days	1.49 (.66, 3.33)	6.78 (2.80, 16.43)***
*Sex* ^d^	F (2,177) = 6.63**	
*Current marks in school*, *on average* ^d^	F (2,177) = 9.54***	
**Energy drinks mixed with alcohol (n = 3309)** ^3^	F (8,158) = 9.35***	
Never	1.00 (Reference)	1.00 (Reference)
Lifetime^a^	2.55 (1.39,4.70)**	1.71 (.59, 4.90)
1–2 times^b^	1.12 (.72, 1.74)	2.35 (1.49, 3.69)***
3–5 times^b^	1.16 (.53, 2.54)	2.76 (1.20, 6.34)*
6 +^b^	1.80 (.88, 3.69)	7.70 (3.81, 15.59)***
*Sex* ^d^	F (2, 164) = 6.08**	
*Current marks in school*, *on average* ^d^	F (2, 164) = 8.78***	

Notes:

^(1)^ Listwise deletion resulted in the following n’s: never (n = 7780), former (lifetime excluding past 12 months) TBI (n = 1526) and recent (past 12 months)TBI (n = 593);

^(2)^ Listwise deletion resulted in the following n’s: never (n = 7801), former TBI (n = 1556), and recent TBI (n = 612);

^(3)^ Listwise deletion resulted in the following n’s: never (n = 2486), former TBI (n = 555), recent TBI (n = 268), this variable was assessed in only one of the two forms of the questionnaires;

In complex samples, df = (# PSUs)—(# strata);

*** significant at P < 0.001;

** significant at P < 0.01;

* significant at P < 0.05 (two-tailed);

^a^ Lifetime, excluding past 12 months;

^b^ Past 12 months;

^c^ Occasionally/Frequently;

^d^Covariate

In general odds ratios for alcohol consumption, energy drinks and energy drinks mixed with alcohol were lower for predicting former TBI compared to recent TBI. Specifically, relative to those not sustaining a TBI, odds ratios for a former TBI were significant for infrequent to occasional/frequent alcohol consumption in the past 12 months (aORs = 1.29 to 1.54, respectively), energy drinks consumption in the past 12 months, between 2 to 4 times in past 7 days and once during the past 7 days and (aORs = 1.55, 2.24, and 2.34, respectively), and energy drinks consumption with alcohol in lifetime but not in the past 12 months (OR = 2.55). Compared to abstainers, infrequent to occasional/frequent drinkers had adjusted odds of 1.98 to 3.65 times higher to have sustained a recent TBI than not. Adjusted odds of sustaining a recent TBI were positively related to frequency of energy drink consumption rising from 2.04 (past 12 month consumed at least once) to 6.78 (5+ energy drinks consumed in the past 7 days). This increasing pattern was also observed for consumption of energy drinks mixed with alcohol on the odds of sustaining a recent TBI (adjusted odds ratios increasing from 1.71 among those who reported lifetime consumption to 7.70 among those who reported consuming these drinks 6+ times in the past 12 months).

Three further multinomial logistic regressions reliably distinguished among *recent TBIs that occurred during sport participation* (e.g., team sports) relative to *recent TBIs that occurred as a result of other causes* (fell down by accident, fight, pushed as a result of bullying, motor vehicle accident, bicycle accident, other causes not assessed) (Table 3); alcohol use Wald’s *F (5*,*148)* = 4.48, P < 0.01, energy drinks consumption Wald’s *F (6*,*149)* = 4.91, P < 0.001, and alcohol-energy drink consumption Wald’s *F (6*,*96)* = 2.31, P < 0.05. Relative to those students sustaining a recent TBI due to other mechanisms of injury other than sports, the odds of sustaining a TBI due to injuries that occurred while playing sports (e.g., team sports) was greater among students who consumed at least one energy drink in the past year (aOR = 2.18) than students who never consumed energy drinks. Relative to those students sustaining a recent TBI due to sports injuries, the odds of sustaining a recent TBI due to injuries that occurred while not being engaged in playing sports (riding a bike, falling, etc.) were greater among students who consumed five or more energy drinks in the past 7 days (aOR = 6.36; CI: 1.48, 27.42) than students who never consumed energy drinks.

**Table 3:** Multiple logistic regression analyses predicting *recent (past 12 months) TBI due to sports injuries* relative to *recent TBI due to other mechanisms of injury* by fitting alcohol use, energy drinks, energy drinks mixed with alcohol, respectively, while controlling for sex and academic performance. The Table displays adjusted Odds Ratios (OR) and design adjusted 95% CI for TBI, 2013 OSDUHS.

	Recent TBI due to sports injuries vs. Recent TBI due to other mechanisms of injury aOR (95% CI)
*Model*	*F 5*,*148) = 4*.*48* **
*Alcohol Use (n = 552)* ^1^	*F (3*, *150) = 2*.*25*
Never used	1.00 (Reference)
Lifetime^a^	0.17 (0.04, 0.69)
Infrequently^b^	0.54 (0.21, 1.38)
Occasionally/Frequently^c^	0.79 (0.37, 1.70)
*Sex* ^d^	*F (1*,*152) = 15*.*13* ***
*Current marks in school*, *on average* ^d^	*F (1*,*152) = 8*.*49* **
*Model*	*F (6*,*149) = 4*.*91* ***
*Energy drinks (n = 570)* ^2^	*F (4*,*151) = 3*.*98* **
Never used^b^	1.00 (Reference)
Past 12 months	2.18 (1.31, 3.63)**
Once in past 7 days	1.13 (.46, 2.76)
2–4 times in past 7 days	1.02 (.24, 4.26)
5 + in past 7 days	0.16 (.04, .68)*
*Sex* ^d^	*F (1*,*154) = 17*.*26* ***
*Current marks in school*, *on average* ^d^	*F (1*, *154) = 7*.*15* **
*Model*	*F (6*,*96) = 2*.*31* *
*Energy drinks mixed with alcohol (n = 245)* ^3^	*F (4*,*98) = 0*.*54*
Never	1.00 (Reference)
Lifetime^a^	1.38 (.20, 9.66)
1–2 times^b^	1.99 (.57, 6.93)
3–5 times^b^	.68 (.16, 2.93)
6 +^b^	.73 (.26, 2.08)
*Sex* ^d^	*F (1*,*101) = 4*.*48* *
*Current marks in school*, *on average* ^d^	*F (1*, *101) = 9*.*44* **

Notes:

^(1)^ Listwise deletion resulted in the following n’s: other causes of recent (past 12 months) TBI (n = 259), and recent TBI due to sports (n = 293);

^(2)^ Listwise deletion resulted in the following n’s: other causes of recent (past 12 months) TBI (n = 268), and recent TBI due to sports (n = 302);

^(3)^ Listwise deletion resulted in the following n’s: other causes of recent (past 12 months) TBI (n = 117), and recent TBI due to sports (n = 128);

In complex samples, df = (# PSUs)—(# strata);

*** significant at P < 0.001;

** significant at P < 0.01;

* significant at P < 0.05 (two-tailed);

^a^ Lifetime, excluding past 12 months;

^b^ Past 12 months;

^c^ Occasionally/Frequently;

^d^Covariate

## Discussion

A primary finding of the present investigation is that recent (past year) as well as former (lifetime, but not in the past 12 months) TBI was associated with the consumption of both alcohol and energy drinks consumption. Recent TBI was also associated with the consumption of the mixture of energy drink and alcohol, but this association was not evident for alcohol-energy drink consumption and former TBI. To our knowledge, this is the first study to investigate the use of energy drinks, and energy drinks mixed with alcohol, among adolescents who reported TBI, and evaluate these predictors in relation to sport injuries relative to other mechanisms of injury among teenagers. A secondary finding of the present investigation is that recent TBI was more strongly related to consuming alcohol, energy drinks and energy drinks and alcohol mixed. Consumption of these substances may be a coping mechanism to deal with the effects of TBI, or they may predispose adolescents to TBI, or both [34–35]. These results are consistent with recent studies that have linked the use of energy drinks by high school and college students with risk taking behaviours and increased physical injuries [24,34–35]. The potential for negative synergistic effects of combined TBI and substance problems showed by previous research, including impact on academic performance, subsequent limited vocational options after high-school, and more risk-taking behaviours suggests that this should be a priority for future studies [10–11,24,36].

The association between TBI and energy drink consumption may not be surprising since the advertising of energy drinks often uses both team and non-team sports scenarios to promote these products [17–24]. Team sports remain the main mechanism of TBI among adolescents compared with all other mechanisms of injury reported [1–5]. However, the current study identifies significant positive associations between recent TBIs sustained during sports participation relative to those that sustained a recent TBI due to other causes and consumption of energy drinks (at least once in the past year) when compared to abstainers, and between recent TBI sustained during any other activities but sports relative to those that sustained a recent TBI due to sports participation and consumption of energy drinks (5+ in the past 7 days). Adolescent energy drink use may be more positively associated with sensation seeking while engaging in sports activities as shown to be the case among one adolescents and several young adult users studies [24,36,37,38], but also head injuries associated with biking, motor vehicle accidents, falling and fighting. Indeed, results from a 2011 cross-sectional study of adolescents conducted in Ontario, Canada, showed a positive association between self-reported physical injuries reported to a medical doctor in the past 12 months (reported by 42% of the adolescents in the sample) as well as sensation seeking (reported by 16% of the adolescents in the sample) and energy drinks consumption as reported in past 7 days, as well as past year (but not in the past 7 days) [24]. Among those who reported high sensation seeking and those who reported being injured and treated in the previous year, almost 39% and 28%, respectively, had consumed energy drinks in the previous week compared to almost 16% and 13% of those who did not report high sensation-seeking and injury, respectively. To our knowledge, this is the first evidence-based data to link sports TBI with consumption of energy drinks among teenagers in a population-based sample. These results, however, may not be surprising. Evidence in recent years points to increased risk for energy drinks following participation in a variety of school and intramural sports [20,21].

One in five Ontario students in grades 7 through 12 in this Canadian province-wide school survey reported a lifetime TBI, and 6% sustained a TBI in the previous year. Estimated lifetime prevalence of TBI in this population showed no change between 2011 and 2013, indicating that TBI remains a prevalent health concern among youth [4]. The relationships among TBI, alcohol use and poor academic performance and its stable prevalence between 2011 and 2013 underscores the need for prevention [2,4,5,7].

This work is not without limitations, which include possible bias related to self-report procedures, the exclusion of groups who might be at higher or lower risk for TBI (institutionalized) and preclusion of causal inferences. Although our response rate was good and our data did not show evidence of appreciable bias [27], potential non-response bias cannot be ignored. Another limitation of our study is the lack of information regarding the temporal sequence between the occurrence of TBI and the consumption of energy drinks and alcohol we observed. Based on our data we cannot establish causality. The consumption of energy drinks and alcohol, either singly or in combination, may represent a coping mechanism to deal with the effects of TBI, or predisposing factors for adolescent TBI, or all may result from common underlying causes such as increased propensity for risk taking. Lastly, our operational definition excluded milder forms of traumatic brain injury that leaves the individual confused or dazed without loss of consciousness, or resulted in loss of consciousness for less than 5 minutes. Several studies have shown that among surveyed brain injured adults whose injury was not accompanied by loss of consciousness or their loss of consciousness lasted less than 5 minutes, as many as 40% to 66% of them also reported having had a history of problem drinking behavior, use of illegal drugs or both [39–41]. Additional research to assess the associations between such milder forms of TBI and the correlates we investigated here is needed, as well an investigation of the role of multiple episodes and severity of TBI in these associations.

Future studies should examine the context in which energy drinks are used (e.g., in sports to help adolescents stay alert, to study for school, etc.), and the extent to which they represent a contributor to TBI, a consequence of TBI, or are a result of common underlying factors. Studies on energy drinks and adolescent behaviour support the idea that energy drinks consumption may compound the natural tendency to engage in risk taking behaviours, although few possible mechanisms for this association have been demonstrated [19,24,35]. One behavioural and imaging study suggests that TBI can diminish the ability to understand the future consequences of one’s actions, but more research is needed to fully understand this relationship [41].

This is the first study to assess the relationship between energy drink use, consumption of energy drinks mixed with alcohol and TBI in an adolescent population. To our knowledge this is also the first investigation to examine the hypothesized link between TBI caused by sport injuries and consumption of alcohol, energy drinks and mixing alcohol with energy drinks among adolescents. The magnitude of the prevalence estimates and the associated risks identified within this representative sample support suggestions to reduce media advertising promoting the use of energy drinks in sports, and highlight the need improve understanding of how these measures are linked.
